# Comparative Analysis of Ovarian Cancer Tumor-Infiltrating T Cells with Those in Melanoma and before and after PD-1 Blockade

**DOI:** 10.1158/2767-9764.CRC-26-0011

**Published:** 2026-11-12

**Authors:** Ilaria Colombo, Ksenia Meteleva, Simone C. Stone, Anjelica Hodgson, Nicolas Jacquelot, Aras Toker, Azin Sayad, Jehan Vakharia, Valentin Sotov, Christine Tran, Hal K. Berman, Stephenie Prokopec, Giselle Boukhaled, Lisa Wang, Pamela Soberanis Pina, Neesha Dhani, Amit M. Oza, Marcus O. Butler, Lillian L. Siu, Ben X. Wang, Pamela S. Ohashi, Stephanie Lheureux

**Affiliations:** 1 Oncology Institute of Southern Switzerland, Ente Ospedaliero Cantonale (EOC), Bellinzona, Switzerland.; 2 Division of Medical Oncology and Hematology, Princess Margaret Cancer Centre, University Health Network, Toronto, Canada.; 3 Department of Immunology, University of Toronto, Toronto, Canada.; 4 Tumor Immunotherapy Program, Princess Margaret Cancer Centre, Toronto, Canada.; 5 Department of Laboratory Medicine and Pathobiology, University of Toronto, Toronto, Canada.; 6 Department of Biochemistry and Molecular Biology, Cumming School of Medicine, University of Calgary, Calgary, Canada.; 7 Riddell Centre for Cancer Immunotherapy, Arnie Charbonneau Cancer Research Institute, Arthur J.E. Child Comprehensive Cancer Centre, Calgary, Canada.; 8 Department of Microbiology, Immunology and Infectious Disease, Cumming School of Medicine, University of Calgary, Calgary, Canada.; 9 Alberta Children’s Hospital Research Institute, Calgary, Canada.; 10 Calvin, Phoebe, and Joan Snyder Institute for Chronic Diseases, University of Calgary, Calgary, Canada.; 11 Princess Margaret Cancer Centre, University Health Network, Toronto, Canada.; 12 Department of Biostatistics, Princess Margaret Cancer Centre, Toronto, Canada.

## Abstract

CD8^+^ T cells are involved in natural tumor immune surveillance and are known to play a critical role in mediating responses to programmed cell death protein 1 (PD-1) blockade. High-grade serous ovarian cancer (HGSOC) exhibits intrinsic resistance to immune checkpoint inhibitors (ICI), despite the presence of tumor-infiltrating lymphocytes (TIL) in the tumor immune microenvironment (TIME). In this study, we characterized the phenotype and frequency of TILs in the tumor samples of patients with HGSOC and compared our findings with TILs from melanoma, a tumor highly sensitive to ICI. Melanoma harbored an increased density of CD8^+^ TILs compared with HGSOC. However, CD8^+^ T cells from HGSOC tumor samples expressed higher levels of exhaustion markers such as 2B4 and PD-1 compared with the CD8^+^ melanoma TILs, with evidence for antigen-specific CD8^+^ TILs. Our study demonstrates that despite the presence of CD8^+^ T cells that are phenotypically exhausted, there is limited expansion of CD8^+^ cells after treatment with anti–PD-1 in patients with HGSOC. These data indicate that CD8^+^ T cells from ovarian cancer express markers associated with antigen specificity and are on the exhaustion trajectory yet are unable to contribute to a measurable antitumor response after PD-1 blockade.

**Significance:**

By analyzing the TIME in patient-derived samples of HGSOC and comparing them with melanoma, we identify a lower density of CD8^+^ cells. These cells are characterized by a higher level of immune exhaustion with a poor response after treatment with anti–PD-1.

## Introduction

Ovarian cancer represents the most lethal gynecologic malignancy in developed countries, with 19,680 new cases and 12,740 deaths estimated in 2024 in the United States (1). The majority of patients are diagnosed at an advanced stage, and despite progress in surgical practice, medical treatment, and understanding of tumor biology, prognosis still remains poor, with a 5-year overall survival of ∼45% (2). High-grade serous ovarian cancer (HGSOC) is the most common histologic subtype, representing ∼70% of all epithelial ovarian malignancies (2). Optimal cytoreductive surgery and platinum-based chemotherapy have been, for decades, the milestones for the treatment of ovarian cancer (2). More recently, maintenance treatment with antiangiogenic agents and/or poly (ADP-ribose) polymerase (PARP) inhibitors (in the presence of *BRCA* mutation and homologous recombination deficiency) has significantly improved the outcomes of patients with high-grade ovarian cancer and is now incorporated into the standard-of-care treatment in the first line (2, 3). Nevertheless, many patients will inevitably develop disease recurrence and treatment resistance.

Increasing evidence demonstrates the importance of the tumor immune microenvironment (TIME) in ovarian cancer. The presence of tumor-infiltrating lymphocytes (TIL), particularly a high CD8^+^/regulatory T-cell (Treg) ratio, has been shown to positively correlate with outcomes (4–6). In addition, a higher frequency of B cells colocalized with CD8^+^ T cells has been shown to improve patient survival (5).

Chronic antigen stimulation can lead some tumor antigen–experienced CD8^+^ T cells to exhaustion, which is characterized by reduced proliferative capacity and cytokine production, and increased expression of inhibitory markers, such as programmed cell death protein 1 (PD-1; ref. 7). Notably, PD-1 and programmed cell death ligand 1 (PD-L1) inhibitors act on a defined subset of CD8^+^ T cells that are differentiating along the exhaustion trajectory, called progenitors of exhausted T cells (Tpex), which arise in the context of chronic antigen stimulation and mediate antitumor responses after therapy. Tpex precursor population is a long term–persisting subset of PD-1^+^ TCF-1^+^ (T-cell factor 1) CD8^+^ T cells that are able to self-renew and differentiate into PD-1^+^ TCF-negative effector exhausted T cells (Tex; refs. 8–11). Importantly, Tpex cells proliferate in response to anti–PD-1 treatment and give rise to terminally differentiated effector Tpex cells that harbor exhausted features yet are able to mediate effective antitumor responses (8, 12, 13).

Although immune checkpoint inhibitors (ICI) have significantly changed the treatment landscape of different malignancies, disappointing results have been reported in ovarian cancer either as a single agent or in combination with chemotherapy, with or without the VEGF inhibitor bevacizumab (14). However, when combined with antiangiogenic and PARP inhibitors, signs of activity have been reported (15, 16). More recently, the KEYNOTE B96 study demonstrated a statistically significant improvement in progression-free and overall survival with the combination of weekly paclitaxel and pembrolizumab ± bevacizumab in patients with PD-L1 combined positive score ≥1 platinum-resistant ovarian cancer (17). The biological mechanisms underlying this unexpected treatment failure, particularly in contrast to the favorable outcomes reported in the KEYNOTE-B96 study, remain incompletely understood. These positive results may partly reflect the unique immunomodulatory properties of weekly paclitaxel, which has been shown to enhance tumor immunogenicity and promote a more inflamed tumor microenvironment (TME), thereby potentially augmenting the efficacy of PD-1 blockade and patient selection (PD-L1–positive and no more than two prior lines of treatment; ref. 18). To date, no validated predictive biomarkers of sensitivity or resistance to ICI in ovarian cancer have been validated, and the cause underlying the lack of efficacy in the majority of phase III trials has not yet been understood.

To explore the dynamic changes occurring after treatment with PD-1 blockade, we analyzed the tumor tissue of patients with HGSOC treated with anti–PD-1 (pembrolizumab) as part of a basket phase II single-center study enrolling patients with advanced solid tumors (INSPIRE trial, NCT02644369; ref. 19). To further characterize CD8^+^ T cells in HGSOC tumors, we compared our results with a cohort of newly diagnosed HGSOC and a control cohort of patients with melanoma, a tumor known to be highly sensitive to ICI.

## Materials and Methods

### INSPIRE clinical trial design and patient population

One objective of our study was to assess changes in the immune landscape in tumor tissue in patients with HGSOC treated with single-agent pembrolizumab as part of the INSPIRE trial (INSPIRE cohort). Patients underwent tumor biopsy before and during treatment (after two or three triweekly cycles), as well as a mandatory collection of archival tissue.

The protocol has been approved by the Institutional Review Board and conducted according to the Good Clinical Practice Guidelines and the Declaration of Helsinki. All patients provided written informed consent to participate in the INSPIRE trial.

Tumor samples from patients with newly diagnosed HGSOC or melanoma were collected from the University Health Network Cancer Biobank for comparative analysis. All patients provided written informed consent for the use of samples for future research.

### Treatment and assessment

Patients enrolled in the INSPIRE trial received pembrolizumab 200 mg as a 30-minute intravenous infusion on day 1 of each 21-day cycle. Patients continued treatment until documented disease progression or unacceptable toxicity. Radiologic disease assessment was performed according to RECIST 1.1 criteria every 9 weeks. Adverse events were monitored throughout the study and for 30 days after treatment discontinuation and were graded according to the National Cancer Institute Common Terminology Criteria for Adverse Events version 4.03.

### Translational and biomarkers analysis

Multiplex immunohistochemistry (mIHC) analysis has been performed on baseline and on-treatment fresh biopsy samples and on archival tissue collected at the time of diagnosis for the INSPIRE cohort, as well as additional HGSOC and melanoma tumor samples of newly diagnosed patients identified for comparative analysis. The PD-L1 score was assessed on the baseline biopsy according to the total modified proportional score (MPS) based on the QualTek IHC analysis. In addition, patient TIL status was assessed using mIHC, Cytometry by Time-of-Flight (CyTOF), and multicolor flow cytometry.

### Multiplex immunofluorescence

Tissue sections of 5 μm were cut from formalin-fixed, paraffin-embedded blocks and mounted onto positively charged slides. Slides were stained with a panel containing CD3, CD8, CD103, PD-1, 4-1BB, and 4′,6-diamidino-2-phenylindole (DAPI) and a panel with CD3, CD68, PD-L1, IDO1, CD163, DAPI and either pan-cytokeratin (CK) for ovarian tissue slides or a melanoma antibody cocktail (HMB45 + M2-7C10 + M2-9E3 + T311 + SOX10) for melanoma slides, using the OPAL 7-color IHC kit (Akoya Biosciences, cat. #NEL811001KT, RRID:AB_3665660) following the manufacturer’s protocol (Supplementary Table S1). Staining was performed on an intelliPATH FLX autostainer (Biocare Medical). Stained slides were scanned in a Vectra 3 imaging system [Akoya Biosciences Vectra 3 Automated Quantitative Pathology Imaging System (RRID:SCR_025828)]. Regions of interest were defined by a pathologist. Images were analyzed in INFORM software (RRID:SCR_019155; version 2.2.0). Tissues were segmented into tumor and stromal areas based on CK or melanoma antibody cocktail staining. Immune cells within tumor and stromal areas were identified and quantified. Data were normalized by tissue area (cells/mm^2^).

### CyTOF staining protocol, sample acquisition, and analysis

Purified mAbs devoid of carrier proteins were labeled with heavy metals at the Flow and Mass Cytometry Facility, Hospital for Sick Children (Supplementary Table S2). Samples were acquired on a Helios mass cytometer [Fluidigm CyTOF-Helios Mass Cytometer (RRID:SCR_019916)] by the Flow and Mass Cytometry Facility, Hospital for Sick Children. Data were normalized using CyTOF software [Fluidigm CyTOF (RRID:SCR_021055), version 7.0]. Data were manually pregated on CD4^+^ and CD8^+^ T cells (Supplementary Fig. S1).

Samples were acquired in nine batches and transformed using hyperbolic arcsine with cofactor 5. To mitigate technical artifacts, a batch correction module from the cyCombine package was applied to the data (20). The CD4^+^ and CD8^+^ TILs were separately clustered via the default Louvain algorithm of the Seurat package version 5 (RRID:SCR_016341; ref. 21). A clustering resolution of 0.25 was applied, yielding seven CD8 and seven CD4 T-cell clusters. Data were dimensionally reduced for visualization using Uniform Manifold Approximation and Projection for Dimension Reduction (UMAP; RRID:SCR_018217; arXiv 1802.03426). Trajectory analysis was done in OMIQ software using the Wishbone algorithm.

### Multicolor flow cytometry staining protocol, sample acquisition, and analysis

After thawing viably frozen enzymatically digested tumor samples in complete RPMI 1640 media, cells were stained with fluorescent antibodies as described previously (Supplementary Table S3; ref. 22). The data were subsequently analyzed using FlowJo software (RRID:SCR_008520, BD, version 10.8.2). Flow cytometry was not performed on the INSPIRE cohort in the present study because these samples had been previously analyzed by cytometry as part of an earlier study (19). Following the completion of the reported analyses, no residual tumor tissue was available for additional or subsequent profiling in the current study. Cell density measurements (cells per mm^2^) were obtained exclusively from IHC data. As a result, flow cytometry data are presented as relative frequencies.

### Independent HGSOC and melanoma sample selection criteria and sample overlap

Tumor specimens were collected from patients with HGSOC and melanoma undergoing standard-of-care surgical procedures. Patient characteristics are summarized in Table 1. Sample selection for mIHC, CyTOF, and flow cytometry was determined by tumor tissue availability and assay-specific cell input requirements. Initially, 20 melanoma and 24 ovarian cancer samples were considered for mIHC analysis; however, samples were subsequently excluded if they were not treatment-naive or did not have the right histology. For CyTOF and flow cytometry, we selected viably frozen tumors with approximately 10 million cells before thawing, as thawing typically resulted in approximately 50% viable cell loss before staining. Additional cell loss occurred during staining and washing steps.

**Table 1.** Patients’ characteristics of the INSPIRE cohort and of the independent HGSOC and melanoma cohorts.

Cohort	HGSOC INSPIRE *N* = 21	HGSOC naive mIHC *N* = 17	HGSOC naive CyTOF *N* = 12	HGSOC flow *N* = 8	Melanoma mIHC *N* = 11	Melanoma naive CyTOF *N* = 7	Melanoma flow *N* = 6
Gender	​	​	​	​	​	​	​
Female	21 (100%)	17 (100%)	12 (100%)	8 (100%)	2 (18%)	1 (14%)	3 (50%)
Male	—	—	—	—	9 (82%)	6 (86%)	3 (50%)
Age (years)	​	​	​	​	​	​	​
Age (median)	61	62	64	60	60	70	46
Range	36–74	35–84	37–84	45–72	54–80	53–84	40–61
Previous debulking surgery	​	NA	NA	NA	NA	NA	NA
Yes	19 (90%)						
No	2 (10%)						
Germline *BRCA* status	​	​	​	​	NA	NA	NA
Wild type	17 (81%)	9 (53%)	5 (42%)	5 (62%)			
Mutated	4 (19%)	6 (35%)	3 (25%)	—			
VUS	—	1 (6%)	1 (8%)	1 (13%)			
UNK	—	1 (6%)	3 (25%)	2 (25%)			
Platinum-sensitive/resistant (at the time of enrollment)	​	NA	NA	NA	NA	NA	NA
Sensitive	3 (14%)						
Resistant	18 (86%)						
Prior systemic treatment	​	​	​	​	​	​	​
Yes	21 (100%)	—	—	1 (12.5%)	—	—	5 (83%)
No	—	17 (100%)	12 (100%)	5 (62.5%)	11 (100%)	7 (100%)	1 (17%)
UNK	—	—	—	2 (25%)	—	—	—
PD-L1 Score on baseline biopsy^a^	​	NA	NA	NA	NA	NA	NA
Mean	6						
Median	0						
Range	0–30						

Abbreviations: NA, not applicable; UNK, unknown; VUS, variant of unknown significance.

a Total MPS – IHC QualTek.

For CyTOF analysis, 15 treatment-naive HGSOC and 15 melanoma samples, collected at the time of surgery, were initially selected. After thawing, staining, acquisition, and quality control, 12 treatment-naive HGSOC and 7 melanoma samples contained more than 1,500 live CD8^+^ and CD4^+^ T cells and were included for downstream analysis. Samples with fewer than 1,500 live CD8^+^ and CD4^+^ T cells were excluded.

For flow cytometry, 10 HGSOC and 10 melanoma samples were initially selected. When fewer than two million cells were recovered after thawing, priority was given to the innate lymphoid cell staining panel, as previously reported (19), limiting the number of samples available for the T-cell panel. Of note, in our CyTOF analysis, all HGSOC and melanoma samples were treatment-naive (patients had not received any prior lines of treatment before sample collection). For flow cytometry, five HGSOC samples were treatment-naive, one was from a patient who had received prior systemic therapy, and treatment history was unknown for two patients; all patients with HGSOC were ICI-naive. However, five patients with melanoma had received prior therapy, including immune checkpoint inhibition. Despite these differences in treatment history, findings were comparable across cohorts. Where possible, overlapping samples were profiled across multiple platforms to enable cross-platform comparison. For example, in the treatment-naive HGSOC cohort, 10 samples were analyzed using both mIHC and CyTOF; among these, one sample was additionally analyzed by flow cytometry. Similarly, in the melanoma cohort, four samples were analyzed using both mIHC and CyTOF.

### Statistical analysis

For clinical data, results were summarized using mean, median, counts, and proportion. Progression-free survival (PFS; time from registration until documented disease progression or death, whichever occurred first) was reported using the Kaplan–Meier method. Patients were considered evaluable for response if they received at least one dose of pembrolizumab and underwent a radiologic assessment after at least one dose. The mIHC cell density data were log_10_-transformed, and a two-way ANOVA with Bonferroni correction was applied for multiple comparisons. For the CyTOF and flow cytometry data, a nonparametric Mann–Whitney U test was utilized to assess differences between groups. A *P* value < 0.05 was considered statistically significant. Data analysis and graphing of the mIHC, CyTOF, and flow cytometry data were performed using GraphPad Prism software [GraphPad Prism (RRID:SCR_002798), version 9.0].

## Results

### Baseline patient characteristics

From April 2016 to October 2017, 27 patients with advanced, recurrent HGSOC were screened, and 21 were enrolled in the INSPIRE trial. All 21 patients were evaluable for response. Baseline biopsies were performed in all patients, and 13 had paired biopsies. At the time of data analysis, all patients had discontinued treatment with pembrolizumab: 4 patients for toxicities and 17 for clinical or radiologic progressive disease (PD).

Median age at the time of enrollment was 61 (range, 36–74). Seventeen (81%) patients were wild type for germline *BRCA1/2*, and four (19%) carried a pathogenic germline *BRCA* mutation (three patients in *BRCA1* and one in *BRCA2*). The median number of previous lines of systemic treatment was 3 (range, 1–7), and at the time of enrollment, most patients had platinum-resistant recurrent HGSOC (86%, *n* = 18). All the patients had a PD-L1 score assessed on baseline biopsy [total MPS – IHC QualTek] ≤30 (range, 0–30 and median of 0).

Patient characteristics of the INSPIRE cohort and of the independent HGSOC and melanoma cohorts used for mIHC, CyTOF, and flow cytometry comparative analysis are summarized in Table 1 and Supplementary Table S4.

### INSPIRE cohort clinical outcomes

In the INSPIRE cohort, the median number of administered cycles of pembrolizumab was 3 (range, 1–16). Of the 21 treated patients, all were assessable for response according to RECIST 1.1 criteria. Six (24%) patients had stable disease (SD) as the best response, and 15 (76%) had PD. No objective response has been observed (Supplementary Fig. S2). No difference has been observed according to PD-L1 expression (assessed on archival tissue) and response to treatment [PD-L1–negative group (MPS, 0): 30% SD and 70% PD; PD-L1–positive group (MPS, ≥1): 27% SD and 73% PD]. Two patients achieved durable SD with shrinkage of target lesions: One patient with platinum-resistant disease carrying a germline *BRCA2* mutation received nine cycles of pembrolizumab with a reduction of 14.7% of the sum of target lesions, and one patient with *BRCA* wild-type platinum-resistant HGSOC received 16 cycles of treatment with a reduction of 25.7% of the target lesion. After a median follow-up of 16.24 months (range, 2.30–49.53), the median PFS was 1.8 months [95% confidence interval, 1.37–1.97]. The safety profile was consistent with the known safety profile of pembrolizumab.

### Treatment-naive HGSOC has a lower density of PD-1^+^ CD8^+^ T cells

To understand the extent of CD8^+^ T-cell infiltration in treatment-naive HGSOC tissues and their spatial location, we profiled tumor tissue sections using mIHC. TILs in patients with newly diagnosed and treatment-naive HGSOC (*n* = 17) were compared with those from patients with melanoma (*n* = 20).

**Figure 1.**
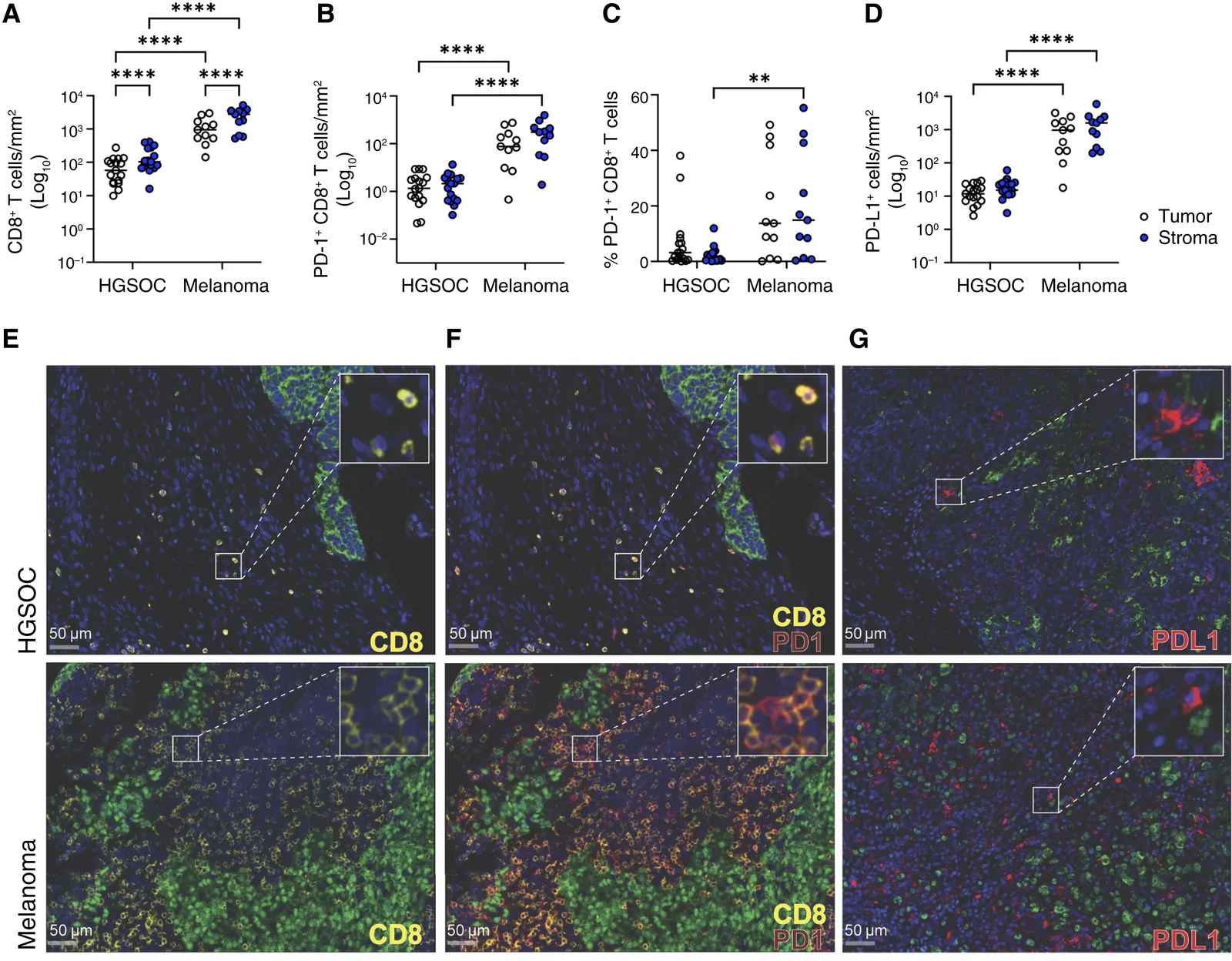
Treatment-naive HGSOC tumors have a lower density of CD8^+^ T cells than melanoma tumors. **A–D,** Melanoma antibody cocktail (melanoma) and pan-CK (HGSOC) staining were used to define tumor/epithelium and stromal areas. **A,** Density of CD8 T cells (CD3^+^CD8^+^) log_10_-transformed. **B,** Density of PD-1^+^ CD8^+^ T cells (CD3^+^CD8^+^PD-1^+^) log_10_-transformed. **C,** Frequency of PD-1^+^ CD8^+^ T cells. *N* = 17 HGSOC/11 melanoma. **D,** Density of total cells expressing PD-L1, log_10_-transformed. **E–G,** Representative mIHC images with a 50 μm scale bar. Insets show selected regions magnified 3.5×. **E,** Representative mIHC images of CD8, (**F**) CD8 and PD-1, and (**G**) PD-L1 staining in HGSOC (top) and melanoma (bottom). All images show DAPI (blue), melanoma antibody cocktail/pan-CK (green), and CD8, PD-1, or PD-L1 staining, as indicated in the bottom right of each image. **, <0.003; ****, <0.0001, two-way ANOVA with Bonferroni correction applied for multiple comparisons.

CD8^+^ T cells were distributed throughout the tumor tissues, with a higher accumulation in the stromal versus intratumoral areas in both HGSOC and melanoma tissues (Fig. 1A). When comparing CD8^+^ T-cell density in melanoma tissues, HGSOC tissues were not heavily infiltrated by CD8^+^ T cells (Fig. 1A and E). Melanoma tumors are known to be highly infiltrated by TILs and are considered hot, which aligns with our findings (23).

Next, we examined the expression of PD-1 in CD8^+^ T cells in treatment-naive HGSOC and melanoma (Fig. 1B and C). HGSOC tumors had a lower density of CD8^+^ T cells positive for PD-1 (Fig. 1B and F). To confirm, we normalized the density of PD-1^+^ CD8^+^ T cells by the total density of CD8^+^ T cells. HGSOC showed a lower frequency of PD-1^+^ CD8^+^ T cells in the stromal compartments versus melanoma (Fig. 1C). We also examined these sections for the expression of PD-L1. Melanoma tumors had a higher density of cells expressing PD-L1 (Fig. 1D and G). PD-L1 expression can be upregulated by interferon γ (IFNγ), secreted by T cells after activation (24). The higher density of CD8^+^ T cells in melanoma tumors could have led to an increased production of IFNγ, which in turn resulted in higher expression of PD-L1. In fact, there was a positive correlation between the CD8^+^ T-cell infiltration and a higher expression of PD-L1 in our treatment-naive melanoma but not in the HGSOC cohort (Supplementary Fig. S3).

### Anti–PD-1 treatment does not increase CD8^+^ T-cell infiltration in HGSOC

To explore changes in CD8^+^ T-cell density and PD-1 expression in patients with HGSOC treated with pembrolizumab (INSPIRE cohort), we performed mIHC on samples collected at the time of diagnosis (archival), prior to ICI treatment (baseline), and during treatment (cycle 2 or 3).

**Figure 2.**
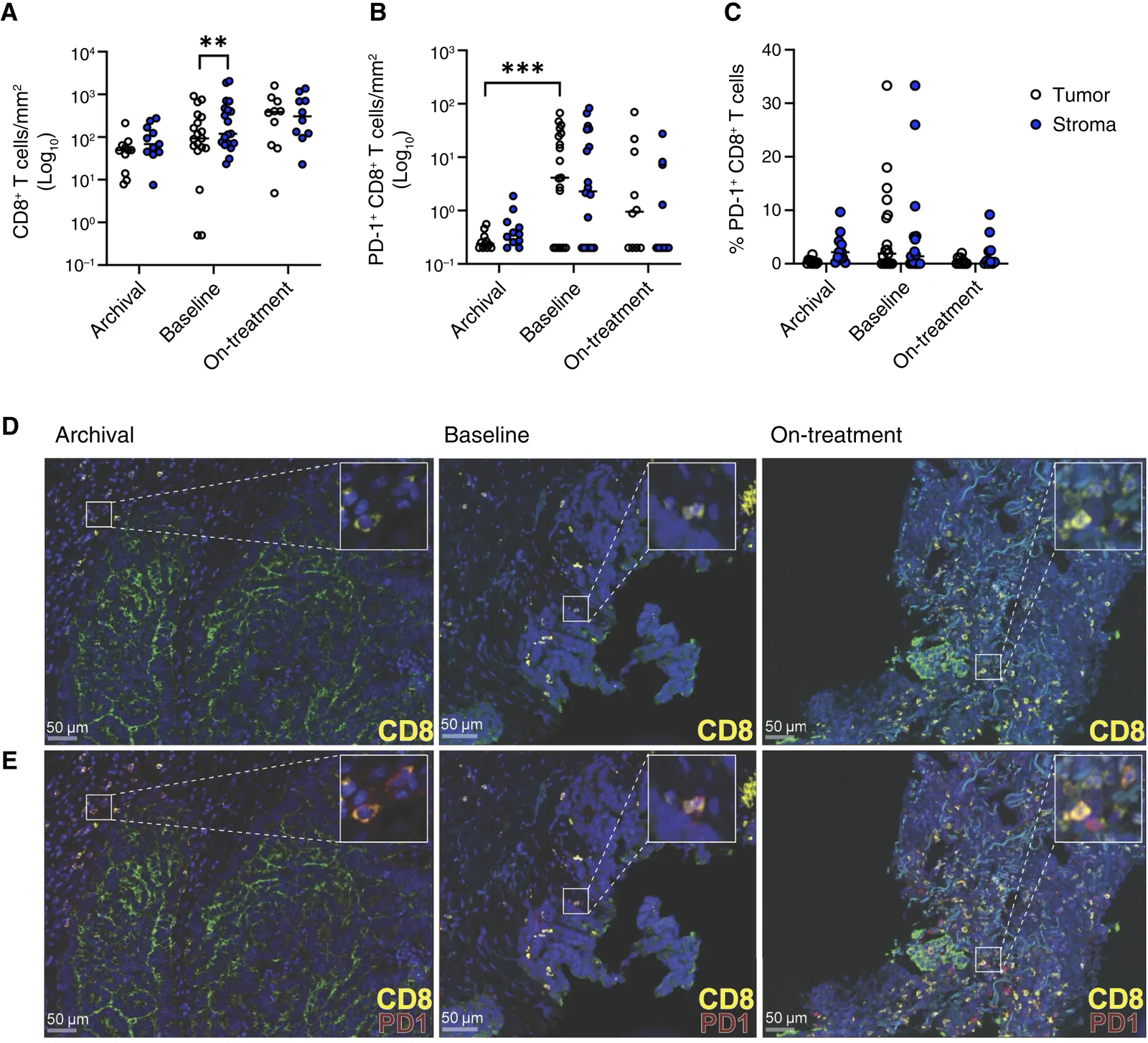
Pembrolizumab did not increase CD8^+^ T-cell density in INSPIRE HGSOC tumors. **A–C,** Pan-CK staining was used to define tumor/epithelium and stromal areas. **A,** Density of CD8 T cells (CD3^+^CD8^+^) log_10_-transformed. **B,** Density of PD-1^+^ CD8^+^ T cells (CD3^+^CD8^+^PD-1^+^) log_10_-transformed. **C,** Frequency of PD-1^+^ CD8^+^ T cells. *N* = 11 archival/19 baseline/10 on-treatment. **D** and **E,** Representative mIHC images with a 50 μm scale bar. Insets show selected regions magnified 3.5×. **D,** Representative mIHC images of CD8 and (**E**) CD8 and PD-1 staining in samples from the same patient at the treatment time points indicated above each image. All images show DAPI (blue), pan-CK (green), and CD8 and/or PD-1 staining, as indicated in the bottom right of each image. **, <0.0027; ***, <0.001; ****, <0.0001, two-way ANOVA with Bonferroni correction applied for multiple comparisons.

Baseline and on-treatment HGSOC samples showed increased but not statistically significant infiltration of CD8^+^ T cells versus archival samples (Fig. 2A and D). Baseline samples had a higher intratumoral density of CD8^+^ T cells positive for PD-1, the target of pembrolizumab (Fig. 2B, C, and E). Importantly, those patients received several lines of treatment prior to pembrolizumab, and our finding likely reflects the TIME evolution.

To examine possible effects of pembrolizumab on CD8^+^ T-cell density, we compared baseline with on-treatment samples. There was a slight but not statistically significant increase in CD8^+^ T-cell density after treatment, including paired samples (Fig. 2A; Supplementary Fig. S4). However, the density of these cells in HGSOC tumors was still not comparable with that of melanoma, as melanoma had a fourfold higher CD8^+^ T-cell density than HGSOC during treatment (Fig. 1A).

### CD8^+^ TILs from treatment-naive HGSOC and melanoma show similar exhaustion profiles

**Figure 3.**
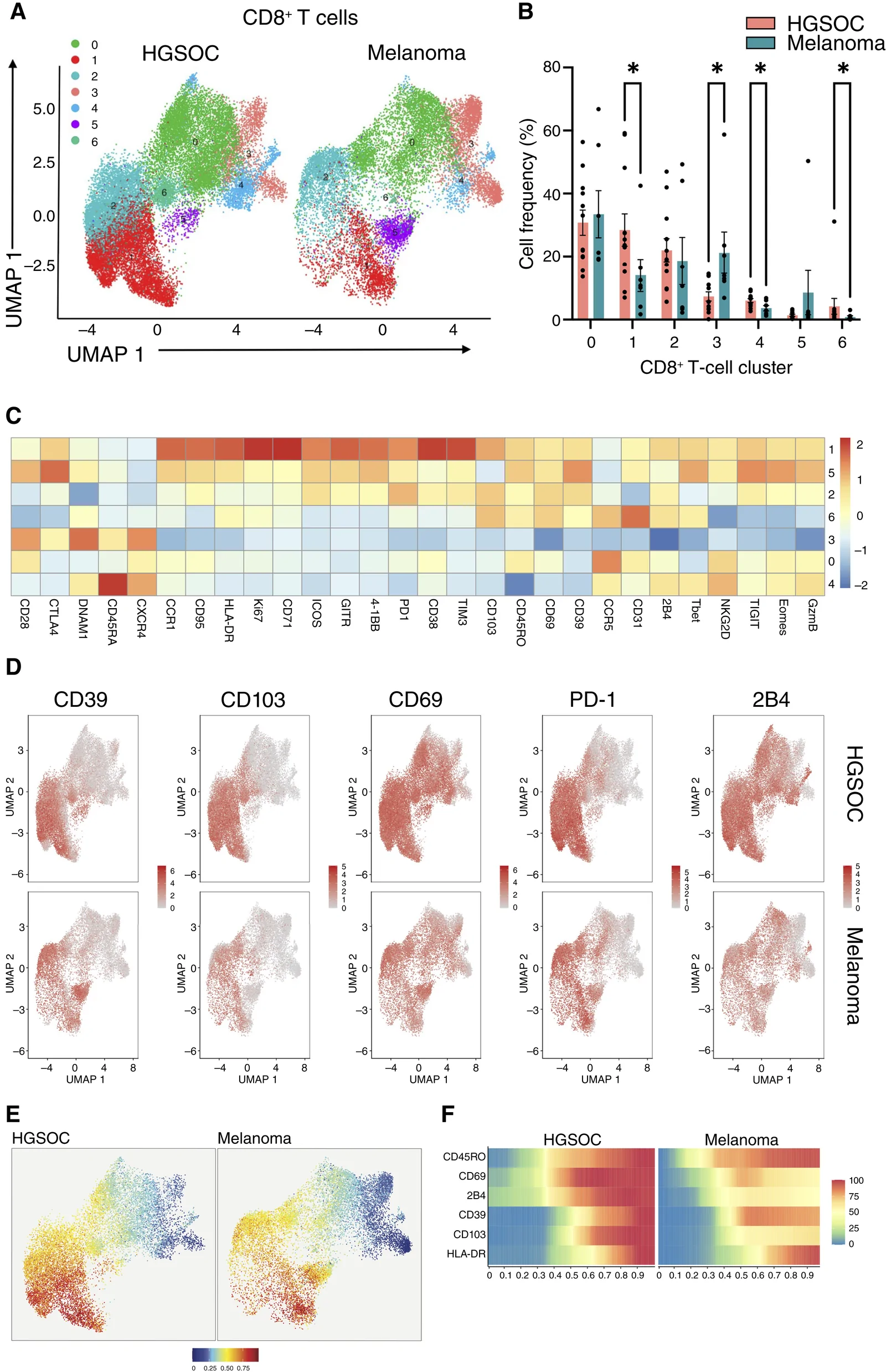
CD8^+^ T cells from treatment-naive HGSOC and melanoma are found in similar clusters. Enzymatically digested, viably frozen HGSOC (*n* = 12) and melanoma (*n* = 7) tumor samples were stained with metal-conjugated antibodies for mass cytometry to characterize the infiltrating CD8^+^ T-cell population. Samples were acquired in batches on a Helios mass cytometer. After CyTOF data were filtered using Gaussian discrimination parameters, viable CD45^+^ CD3^+^ TCRγδ^−^ CD8α^+^ cells were clustered with Seurat (resolution = 0.25) and visualized using a dimensionality reduction algorithm, UMAP. **A,** UMAP of HGSOC and melanoma CD8^+^ TILs, colored by the identified seven clusters. **B,** Frequency of CD8^+^ TILs per cluster in each tumor type, calculated as a percentage of total CD8^+^ TILs per sample. *, 0.05, Mann–Whitney U test without multiple test adjustment. **C,** Heatmap of marker expression per cluster. **D,** Feature plots for selected markers, as indicated at the top of each plot. **E,** UMAP showing Wishbone trajectory with pseudotime score. **F,** Heatmap of selected marker expression in HGSOC and melanoma along the trajectory.

**Figure 4.**
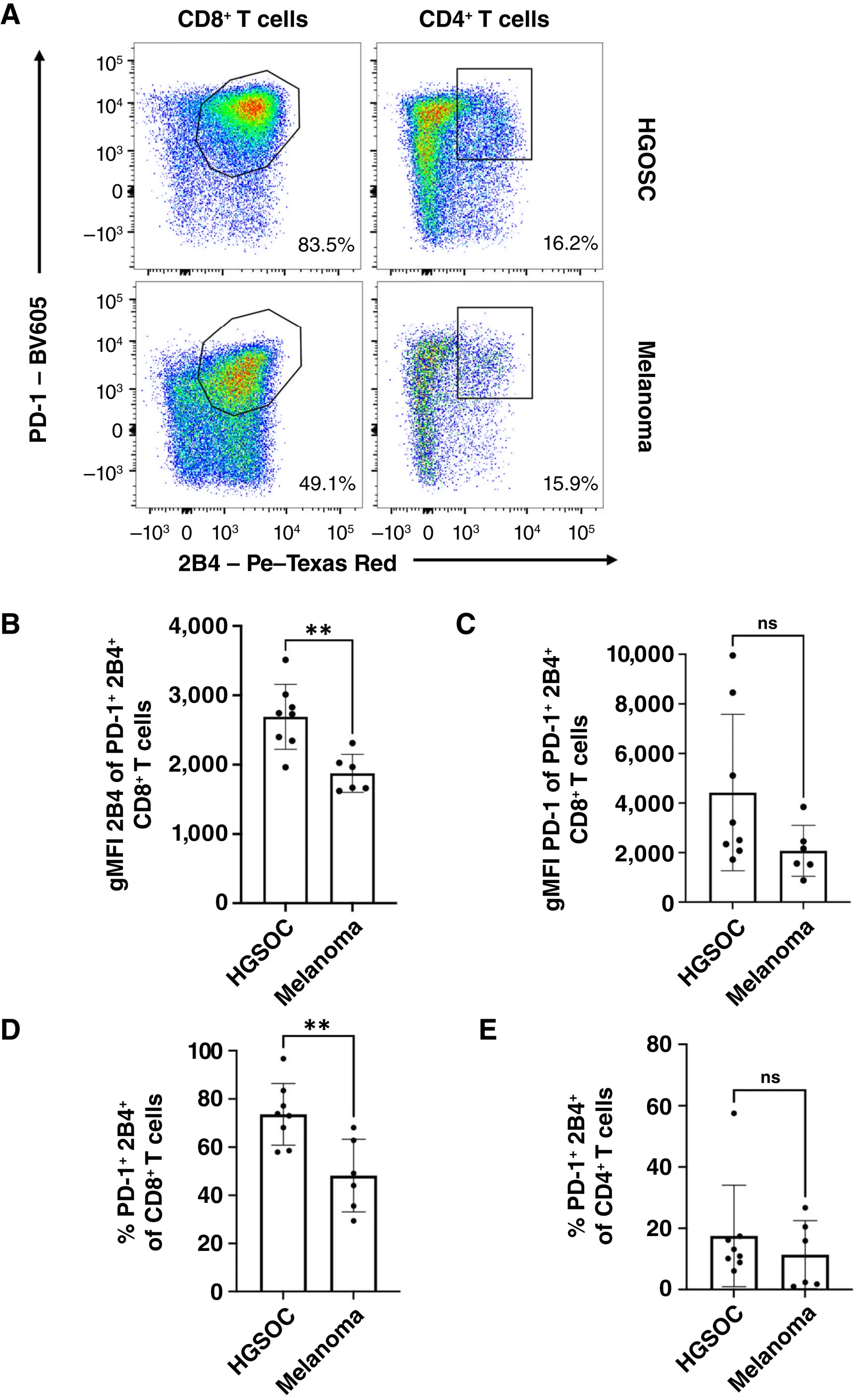
HGSOC tumor samples have CD8^+^ TILs with high expression of 2B4. Enzymatically digested, viably frozen HGSOC (*n* = 8) and melanoma (*n* = 6) tumor samples were stained with fluorophore-bound antibodies for multicolor flow cytometry to characterize the infiltrating CD8^+^ T-cell populations. **A,** Representative PD-1^+^ 2B4^+^ CD8^+^ (left) and CD4^+^ T cell (right) staining and population percentages in HGSOC (top) and melanoma (bottom). **B,** 2B4 expression levels on PD-1^+^ 2B4^+^ CD8^+^ T cells in HGSOC and melanoma as measured by MFI. **C,** PD-1 expression levels on PD-1^+^ 2B4^+^ CD8^+^ T cells in HGSOC and melanoma as quantitated by MFI. **D,** Percentage of PD-1^+^ 2B4^+^ CD8^+^ T cells in HGSOC and melanoma. **E,** Percentage of PD-1^+^ 2B4^+^ CD4^+^ T cells in HGSOC and melanoma. ns, *P* > 0.05; **, *P* < 0.01, Mann–Whitney U test without multiple test adjustment.

As treatment-naive HGSOC tumors exhibited a lower density of CD8^+^ T cells versus melanoma tumors, we queried whether these HGSOC TILs were bystander T cells that were not tumor-reactive or whether they were potentially antigen-specific and expressed typical markers of exhaustion. We profiled CD8^+^ and CD4^+^ T cells from samples of patients with treatment-naive HGSOC and melanoma by CyTOF (Figs. 3 and 4; Supplementary Figs. S1 and S6).

We manually pregated on CD8^+^ and CD4^+^ TILs, excluding Tregs (Supplementary Fig. S1), and assessed the differences associated with the T-cell phenotypes by unsupervised clustering and the dimensionality reduction algorithm UMAP on specimens from each tumor type (Figs. 3 and 4; Supplementary Fig. S6). This analysis revealed seven unique CD8^+^ TIL clusters with differential cell abundances in HGSOC and melanoma tumors (Fig. 3A and B; Supplementary Fig. S5A). HGSOC CD8^+^ TILs had a larger cluster 1, characterized by high coexpression of activation/exhaustion and proliferation markers (Fig. 3C and D). Among these markers are CD103 and CD39, previously shown to represent tumor antigen–specific T cells in various cancers (25, 26). CD103, together with CD69, is also used to identify the nonrecirculating population of tissue-resident memory cells (27). In addition, cluster 1 had high expression of PD-1 and the inhibitory SLAM family receptor CD244 (2B4), previously reported to mark Tex (28, 29).

Treatment-naive melanoma CD8^+^ TILs had a higher abundance of cluster 3, characterized by low expression of activation/exhaustion markers, including PD-1 and CD39—a phenotype consistent with bystander cells (30). These cells are commonly present among TILs, including those from ovarian cancer and melanoma, and are generally nontumor-reactive, with some being virus-specific (10, 31). HGSOC-predominant cluster 4 had lower expression of PD-1, CD39, and markers of tissue resident cells, CD69 and CD103. Additionally, cluster 4 had higher expression of 2B4, T-bet, NKG2D, and granzyme B, likely identifying cells of effector memory phenotype. Cluster 5 showed high expression of CD39, Eomes, T-bet, CTLA-4, TIGIT, and intermediate expression of PD-1 and TIM-3. However, 50% of the CD8^+^ TILs harboring this phenotype came from a single patient with melanoma, whereas other patients had a modest proportion of these cells (Fig. 3B; Supplementary Fig. S5A).

HGSOC-abundant cluster 6 showed consistent expression of tissue resident cell markers CD103 and CD69, similarly to clusters 1 and 2, with high expression of CD31 (Fig. 3B; Supplementary Fig. S5A). This finding is supported by another study that showed there were increased resident memory T cells in HGSOC compared with melanoma (20).

The phenotypic differences between the CD8^+^ TILs present in the treatment-naive HGSOC and melanoma TME suggested that these cells may be differentiating along distinct exhaustion trajectories. We next used the Wishbone trajectory analysis to map the cellular trajectories and the acquisition of activation markers to determine whether the CD8^+^ TILs present in the HGSOC and melanoma TME were differentiating along similar or distinct exhaustion pathways. This analysis revealed that the CD8^+^ TILs from HGSOC and melanoma evolved along the same trajectory (Fig. 3E); however, the cells from melanoma had a reduced expression of CD69, CD39, 2B4, and CD103 (Fig. 3F).

The CD4^+^ TIL compartment analysis showed differential abundances of clusters 2, 3, and 4 between HGSOC and melanoma (Supplementary Fig. S6A and S6B). Similar to the CD8^+^ TIL compartment, cluster 4 of CD4^+^ TILs was positive for Ki67 and PD-1 and was more frequent in HGSOC (Supplementary Fig. S6C). Ki67 is expressed by proliferating cells and, in conjunction with PD-1 expression, could be indicative of tumor-specific response (32). The overall expression of activation/exhaustion markers in CD4^+^ TILs was largely similar in HGSOC and melanoma (Supplementary Fig. S6C and S6D). CD4^+^ TILs also exhibited a less activated phenotype than CD8^+^ TILs in both cancer types.

Our findings showed that HGSOC tumors have a relevant population of CD8^+^ TILs with a highly activated/exhausted phenotype.

### HGSOC CD8^+^ TILs have high expression of 2B4 and PD-1

**Figure 5.**
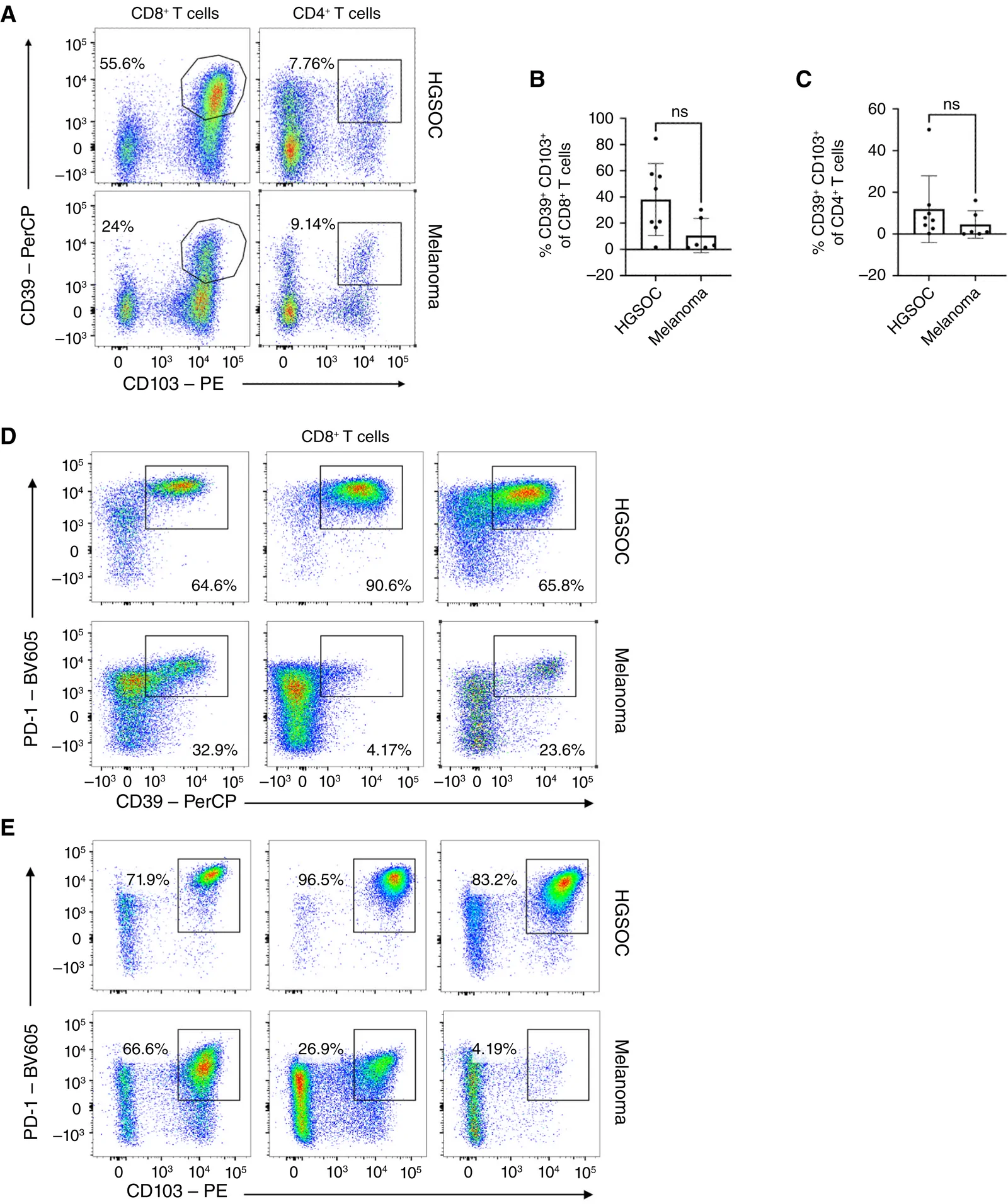
A prominent antigen-specific CD8^+^CD39^+^CD103^+^ population is found in HGSOC. Enzymatically digested, viably frozen HGSOC (*n* = 8) and melanoma (*n* = 6) tumor samples were stained with fluorophore-bound antibodies for multicolor flow cytometry to characterize the infiltrating CD8^+^ T-cell populations. **A,** Representative CD103^+^ CD39^+^ CD8^+^ (left) and CD103^+^ CD39^+^ CD4^+^ (right) staining and population percentages in HGSOC (top) and melanoma (bottom). **B,** Percentage of CD103^+^ CD39^+^ CD8^+^ T cells in HGSOC and melanoma. **C,** Percentage of CD103^+^ CD39^+^ CD4^+^ T cells in HGSOC and melanoma. ns, *P* > 0.05, Mann–Whitney U test. **D,** Cells were gated on CD8^+^ cells, and the CD39^+^ vs. PD-1^+^ are shown with HGSOC (top) and melanoma (bottom) from three representative patient samples; (**E**) PD-1^+^ vs. CD103^+^ analysis is shown from three representative samples of patients with HGSOC (top) and melanoma (bottom).

To further evaluate the differences in marker expression in CD8^+^ and CD4^+^ TILs of patients with HGSOC versus melanoma, we profiled a separate cohort of patients with HGSOC by multicolor flow cytometry and compared results with available melanoma tumors (Figs. 4 and 5; Supplementary Fig. S7). Prior exposure to systemic treatment is detailed in Table 1. Several reports have suggested that elevated 2B4 expression, along with PD-1, TIM-3, and other inhibitory receptors, marks terminally differentiated/exhausted CD8^+^ TILs in human cancers (33, 34). Therefore, we wanted to further determine which cells expressed the marker 2B4 (CD244) and whether this was quantitatively increased in HGSOC compared with melanoma, as suggested by the CyTOF analysis. We found that HGSOC had a significantly higher proportion of 2B4^+^ PD-1^+^ CD8^+^ TILs versus melanoma TILs [mean fluorescence intensity (MFI), 73.61% ± 4.52% vs. 48.17% ± 6.17%, respectively; Fig. 4A and D]. This double-positive population also had significantly higher expression levels of 2B4 in HGSOC versus melanoma (MFI, 2,691 ± 166 vs. 1,876 ± 112, respectively), and although not statistically significant, PD-1 expression was also increased in HGSOC samples compared with melanoma (MFI, 4,423 ± 1,115 vs. 2,072 ± 420, respectively; Fig. 4B and C). Of note, the difference in the abundance of the 2B4^+^ PD-1^+^ population between the two cancers was not conserved in the CD4^+^ TIL compartment (MFI for HGSOC vs. melanoma, 17.51% ± 5.86% vs. 11.37% ± 4.55%, respectively; Fig. 4A and E). CD4^+^ TILs in both HGSOC and melanoma seemed to harbor a similarly low proportion of the 2B4^+^ PD-1^+^ population compared with a double-positive subset found among CD8^+^ TILs in both cancer types (Fig. 4A and E). This indicates that only the CD8^+^ TIL compartment harbors the 2B4^+^ PD-1^+^ population consistent with a highly activated/exhausted phenotype. This is consistent with our CyTOF data, demonstrating that HGSOC CD8^+^ TILs are more exhausted than melanoma TILs.

### HGSOC harbors TILs with a tumor antigen–specific phenotype

As the effective response to immunotherapy is governed by the tumor-reactive T cells in the TIME, we queried whether HGSOC exhibits any differences in the abundance of tumor antigen–specific TILs compared with melanoma. The coexpression of CD39 and CD103 was shown to represent tumor antigen–specific T cells in various cancers (25, 26, 30), and this signature is widely used in the literature as a proxy for the identification of this subset of TILs (25, 35). Flow cytometry analysis showed that HGSOC TILs exhibit a trend toward an increased proportion of CD8^+^ TILs with the tumor antigen–specific phenotype compared with melanoma (Fig. 5A and B). This finding challenges available data in the literature about the limited number of tumor antigen–specific TILs that have been reported in HGSOC (26, 30, 36–38).

When studying the CD4^+^ T-cell compartment, HGSOC had a similar proportion of CD103^+^ CD39^+^ CD4^+^ T cells compared with melanoma (Fig. 5A and C). However, the proportion of CD4^+^ TILs with the tumor antigen–specific phenotype appeared less frequent compared with the CD8^+^ TIL compartment. Nonetheless, this observation is in accordance with the previous findings of CD4^+^ TILs exhibiting lower CD103 expression (35, 39), and therefore, CD39^+^CD103^+^CD4^+^ T cells seem less prevalent than tumor antigen–specific CD8^+^ TILs.

We also determined whether the CD39^+^CD103^+^ CD8^+^ T cells described in Fig. 5A and B overlapped with the Tex population reported in Fig. 4. We gated on CD8^+^ TILs, then analyzed PD-1^+^ versus CD39 and PD-1^+^ versus CD103^+^ populations (Fig. 5D and E). Our flow cytometry analysis showed that in most HGSOC and melanoma samples, CD39 and CD103 are expressed in PD-1^high^ CD8^+^ TILs, indicating that the CD39^+^ CD103^+^ population overlaps with the exhausted CD8^+^ T-cell population. Hence, based on the widely accepted CD103^+^CD39^+^ signature, there is evidence of tumor antigen–specific TILs among the HGSOC TIME, and these putatively tumor antigen–specific TILs would preferentially accumulate in the CD8^+^ as opposed to the CD4^+^ T-cell compartment.

## Discussion

Despite ICIs showing promising results in different malignancies, the activity in ovarian cancer is limited, with a reported objective response rate of only ∼10% when used as a single agent (14). Consistent with other trials (14), we observed limited activity of pembrolizumab, with no objective response in our cohort of patients with HGSOC treated in the INSPIRE trial. To better understand the failure of pembrolizumab in HGSOC, we investigated the baseline features and dynamic changes of the tumor and stromal immune landscape in patients treated with pembrolizumab. We further compared these results with an independent cohort of patients with melanoma, a highly ICI-sensitive disease.

Our mIHC analysis showed that treatment-naive melanoma samples have a higher density of total CD8^+^ T cells with higher PD-1 expression, whereas the relative CD8^+^ T-cell numbers in HGSOC are approximately 15 times lower. Despite the lower frequencies, our CyTOF and flow cytometry analyses showed that CD8^+^ T cells in treatment-naive HGSOC expressed similar markers to treatment-naive or standard care–treated melanoma, including TIM-3, PD-1, TIGIT, and granzyme B, consistent with T cells that are on the exhaustion trajectory (40). Unexpectedly, CD8^+^ TILs from treatment-naive HGSOC expressed high levels of 2B4, suggesting that the HGSOC CD8^+^ population was more exhausted than the melanoma CD8^+^ T cells. However, it remains unclear whether these cells represent terminally exhausted T-cell or Tpex populations. Future studies incorporating TCF-1 expression will be required to distinguish these subsets.

A prevalent opinion in the literature is that only a fraction of CD8^+^ T cells within TIL populations are specific for tumor-expressed antigens (23, 27, 36, 37). In line with the existing literature (28, 39–41), our study has shown the presence of CD8^+^CD103^+^CD39^+^ and conventional CD4^+^CD103^+^CD39^+^ TIL populations in treatment- and ICI-naive HGSOC and melanoma, suggesting that both cancers harbor a detectable pool of antigen-specific T cells. HGSOC had a larger proportion of CD8^+^ antigen-specific TILs compared with melanoma. However, the absolute numbers of antigen-specific T cells in tissue per mm^2^ are likely to be lower, as indicated by our mIHC analysis, which has shown that the total CD8^+^ and PD-1^+^ CD8^+^ T-cell density in treatment-naive HGSOC samples is significantly reduced compared with melanoma samples. Taking into account recent reports in the literature identifying and validating antigenic epitopes in patients with HGSOC (31, 36, 41, 42), our findings support the hypothesis that ovarian cancer is an immunogenic disease with evidence of tumor immune surveillance, especially in the CD8^+^ T-cell compartment. Therefore, the HGSOC TME is not cold, as classically defined. This is consistent with early reports that demonstrate that CD8^+^ T cells correlate with good prognosis in epithelial ovarian cancer (3, 4).

The identification of TILs expressing markers consistent with tumor antigen–specific cells in the TIME of patients with HGSOC by our group and others provides a rationale for the potential improvement of immunotherapy for these patients through different strategies other than ICI (4, 30). Incorporating selective expansion of TILs or T-cell products transduced with specific TCRs against the targets recognized by the patient’s CD8^+^ T cells could provide a personalized therapeutic opportunity to augment cancer cell elimination in HGSOC tumors (31, 41, 43). An important consideration would be the careful selection of T-cell targets that exhibit tumor-restricted or tumor-dominant expression to spare untransformed patient tissue from damage. Another approach that could enhance the priming of tumor-specific T-cell responses against tumor antigens in HGSOC would be the design of personalized cancer vaccines. The use of autologous dendritic cell vaccines pulsed with multiple tumor-restricted antigens or autologous whole-tumor antigen from whole-tumor lysates has already been shown to mobilize neoepitope-specific CD8^+^ T-cell responses in patients with ovarian cancer (30, 43). Vaccines that target tumor neoepitopes showed promise in various cancers as they could elicit the expansion of multiple neoepitope-specific T-cell clones, which can express high-affinity TCRs and thus exhibit high-avidity responses (44–47). Therefore, carefully designed personalized TIL-based therapies and cancer vaccines aimed at harnessing antitumor-restricted T-cell responses could present an encouraging way to attain clinical benefit for patients with HGSOC in view of our biological observations.

The main limitation of our study is the small sample size to assess the status of TILs in the TIME of HGSOC versus melanoma. The extent of our findings should be further validated using larger cohorts, given the heterogeneity of the patient samples profiled. Other limitations include the absence of viably frozen samples directly from the INSPIRE cohort for subsequent cytometry and mIHC analyses. Nevertheless, archival tissue collected at the time of diagnosis was available in the INSPIRE cohort, allowing for a longitudinal assessment of the TIME. Our findings are hypothesis-generating and have provided new insights to support innovative immune strategies in HGSOC.

## Supplementary Material

Supplementary Figure S1 Figure S1. Gating strategy for the CD8+ and CD4+ TILs from HGSOC and MEL samples profiled by CyTOF.

Supplementary Figure S2 Figure S2. Best change in the sum of targets lesions and overall response according to RECIST 1.1.

Supplementary Figure S3 Figure S3. A higher density of CD8+ T cells positively correlates with PD-L1+ cells in MEL

Supplementary Figure S4 Figure S4. Paired analysis of CD8+ T cell in HGSOC tumors after pembrolizumab.

Supplementary Figure S5 Figure S5. Distribution of CD8+ and CD4+ TIL CyTOF clusters per sample.

Supplementary Figure S6 Figure S6. Clusters revealed through high dimensional CyTOF comparison of HGSOC and MEL patient CD8+ and CD4+ TILs.

Supplementary Figure S7 Figure S7: Gating strategy for the CD8+ and CD4+ TILs from HGSOC and MEL samples profiled by the multicolour flow cytometry

Supplementary Table S1 Table S1. Antibody information for the samples profiled by IHC.

Supplementary Table S2 Table S2. Antibody/reagent information for the samples profiled by CyTOF

Supplementary Table S3 Table S3. Antibody information for the samples profiled by multicolor flow cytometry.

Supplementary Table S4 Table S4. Clinical characteristics of melanoma patients

## Data Availability

Raw data for this study were generated at the Princess Margaret Cancer Centre. Derived data supporting the findings of this study are available from the corresponding author upon request.
