# Supplementary material for: Comparative Analysis of Ovarian Cancer Tumor-Infiltrating T Cells with Those in Melanoma and before and after PD-1 Blockade
Source: Cancer Res Commun. 2026 Nov 12;6(11):2655–68. doi: 10.1158/2767-9764.CRC-26-0011 (PMC13757280; doi:10.1158/2767-9764.CRC-26-0011)
Supplement: Supplementary file 1 [file crc-26-0011_supplementary_figure_s1_suppsf1.pdf]

A

HGSOC

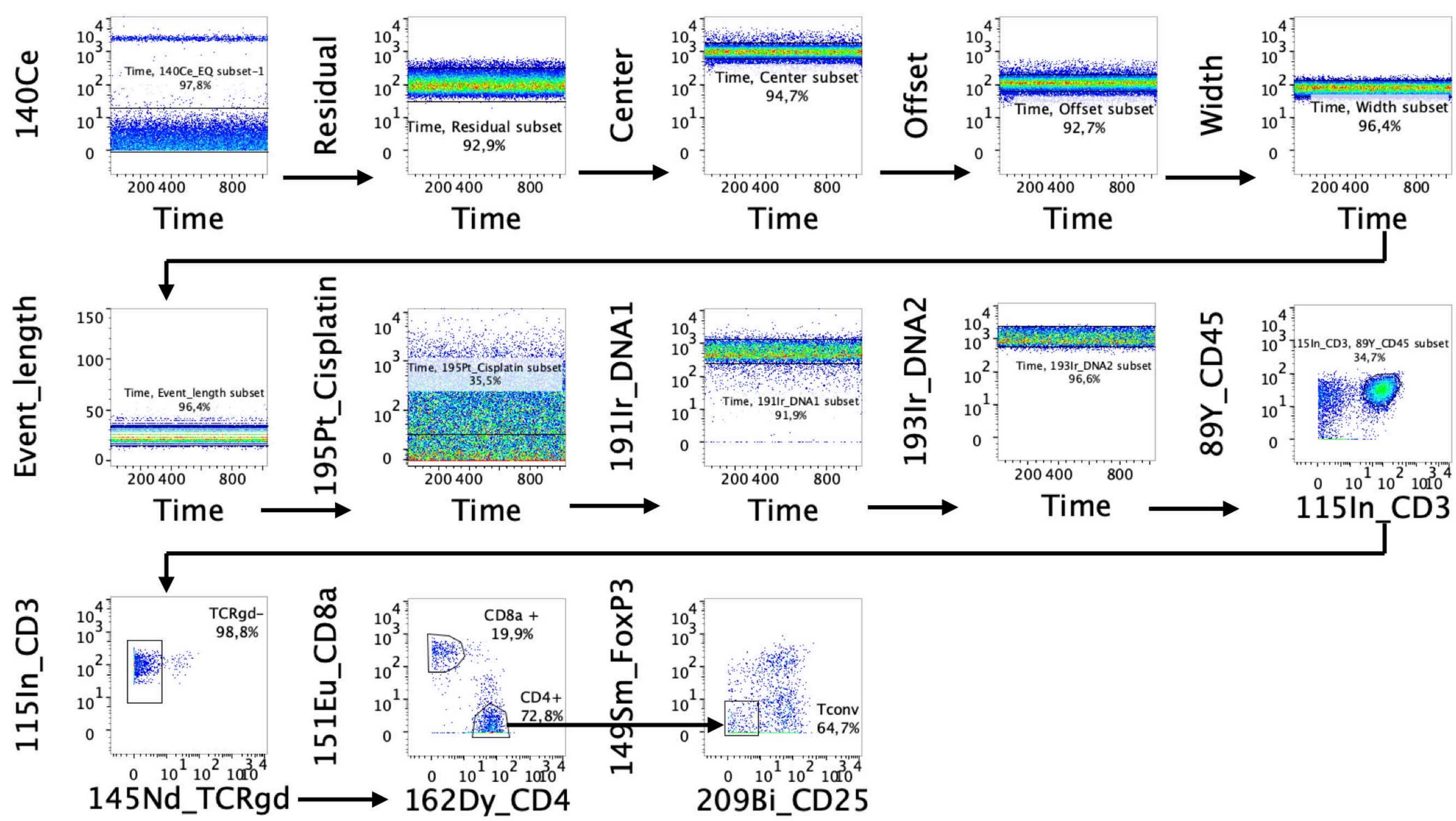
140Ce
Time, 140Ce_EQ subset-1
97,8%
Time
Residual
Time, Residual subset
92,9%
Time
Center
Time, Center subset
94,7%
Time
Offset
Time, Offset subset
92,7%
Time
Width
Time, Width subset
96,4%
Time
Event_length
Time, Event_length subset
96,4%
Time
195Pt_Cisplatin
Time, 195Pt_Cisplatin subset
35,5%
Time
191Ir_DNA1
Time, 191Ir_DNA1 subset
91,9%
Time
193Ir_DNA2
Time, 193Ir_DNA2 subset
96,6%
Time
89Y_CD45
115In_CD3, 89Y_CD45 subset
34,7%
115In_CD3
115In_CD3
TCRgd-
98,8%
145Nd_TCRgd
151Eu_CD8a
CD8a +
19,9%
CD4+
72,8%
162Dy_CD4
149Sm_FoxP3
Tconv
64,7%
209Bi_CD25

B

MEL

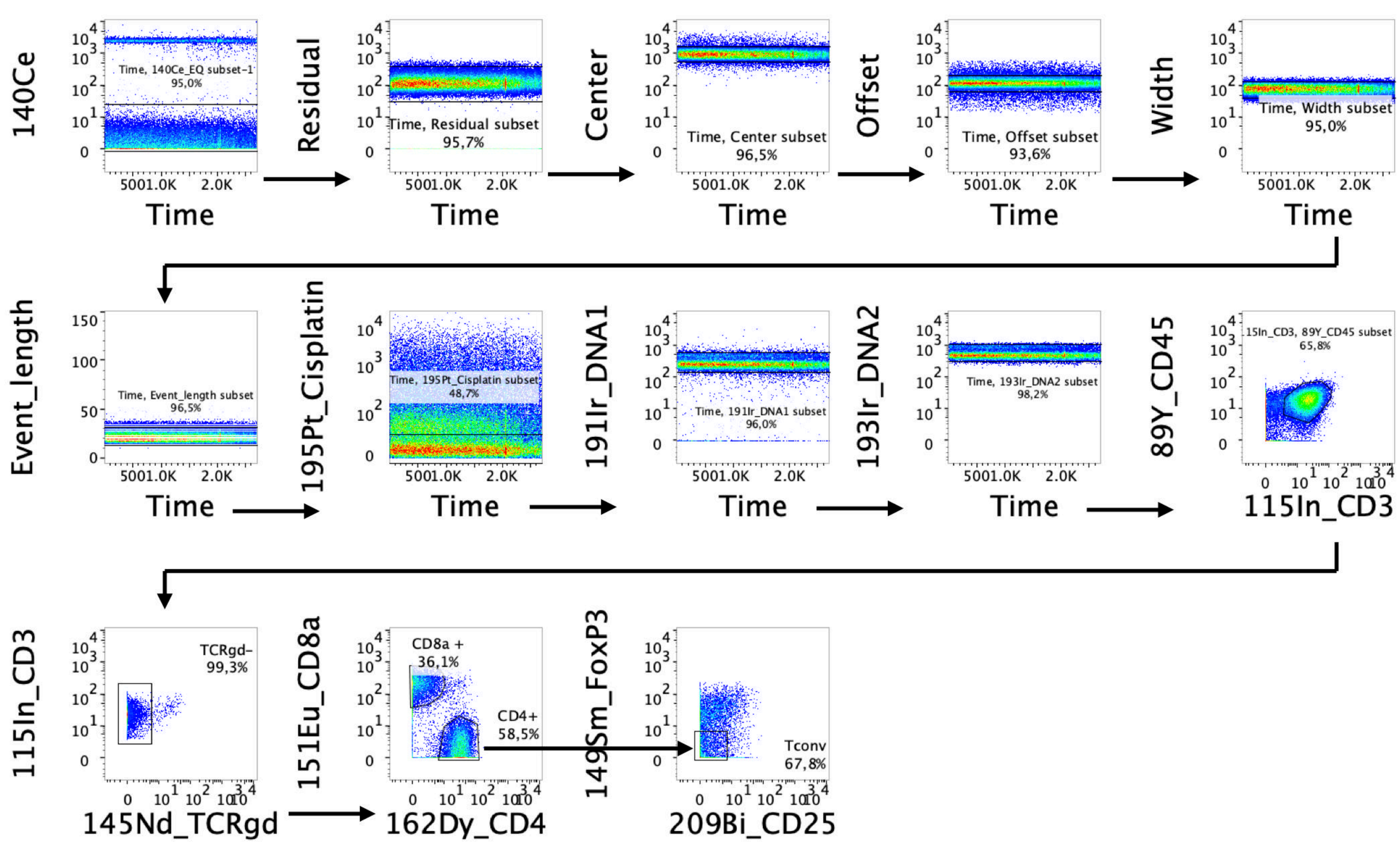
140Ce
Time, 140Ce_EQ subset-1
95,0%
Time
Residual
Time, Residual subset
95,7%
Time
Center
Time, Center subset
96,5%
Time
Offset
Time, Offset subset
93,6%
Time
Width
Time, Width subset
95,0%
Time
Event_length
Time, Event_length subset
96,5%
Time
195Pt_Cisplatin
Time, 195Pt_Cisplatin subset
48,7%
Time
191Ir_DNA1
Time, 191Ir_DNA1 subset
96,0%
Time
193Ir_DNA2
Time, 193Ir_DNA2 subset
98,2%
Time
89Y_CD45
115In_CD3, 89Y_CD45 subset
65,8%
115In_CD3
115In_CD3
TCRgd-
99,3%
145Nd_TCRgd
151Eu_CD8a
CD8a +
36,1%
CD4+
58,5%
162Dy_CD4
149Sm_FoxP3
Tconv
67,8%
209Bi_CD25

Figure S1: Gating strategy for the CD8+ and CD4+ TILs from HGSOC and MEL samples profiled by CyTOF. Using Gaussian discrimination parameters (Time, Residual, Center, Offset, Width, Event length) viability stain (Cisplatin) and DNA intercalators (DNA1 and DNA2), intact, live, singlet cells from a representative HGSOC sample (A) or MEL sample (B) were discriminated from beads (detected in 140 Ce channel) and debris, and gated for the CD45+ CD3+ TCRγδ - CD8α + T cells or CD45+ CD3+ TCRγδ - CD4+ FoxP3- CD25- T cells, as shown.
