# Supplementary material for: Comparative Analysis of Ovarian Cancer Tumor-Infiltrating T Cells with Those in Melanoma and before and after PD-1 Blockade
Source: Cancer Res Commun. 2026 Nov 12;6(11):2655–68. doi: 10.1158/2767-9764.CRC-26-0011 (PMC13757280; doi:10.1158/2767-9764.CRC-26-0011)
Supplement: Supplementary file 2 [file crc-26-0011_supplementary_figure_s6_suppsf6.pdf]

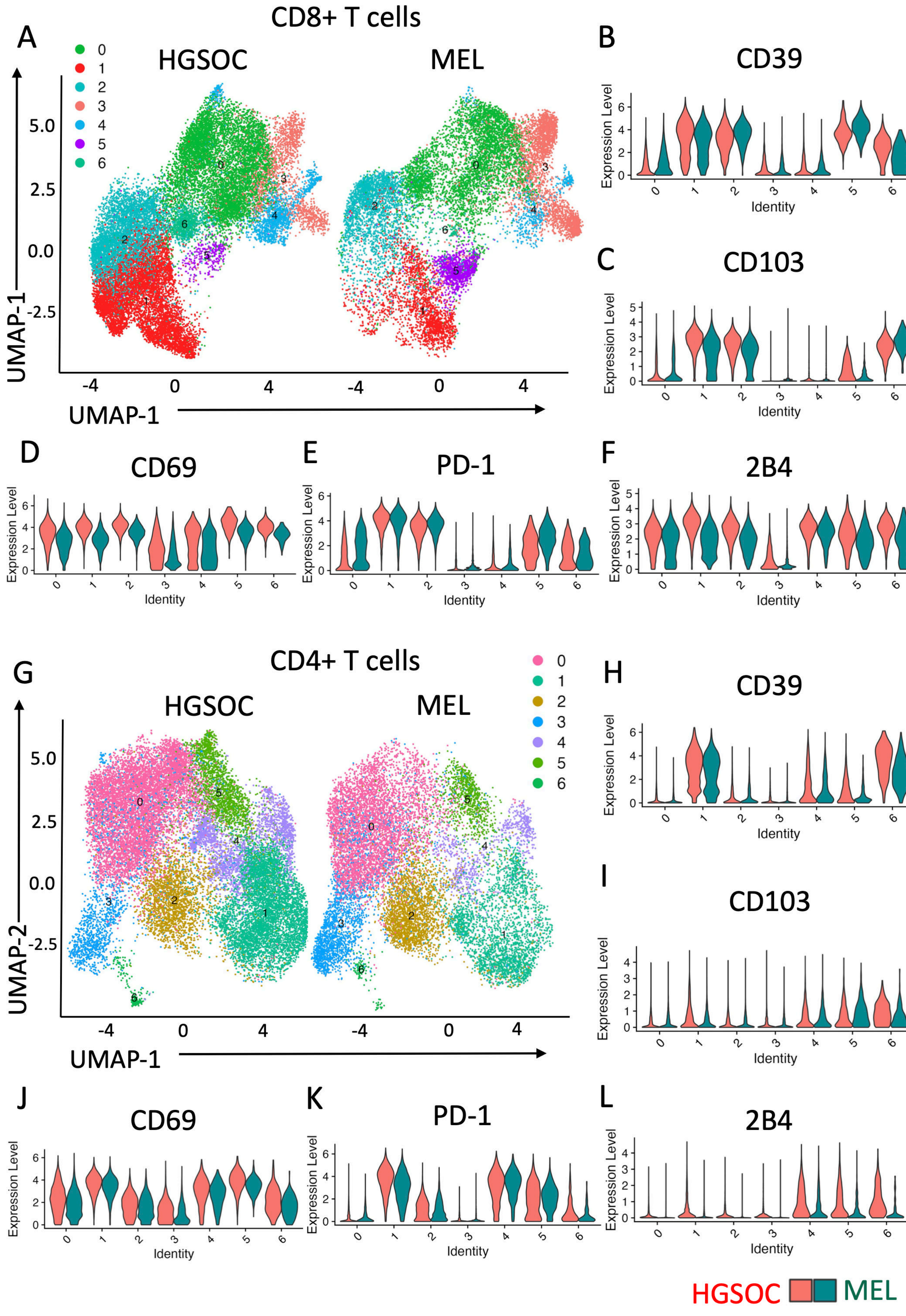

CD8+ T cells
A
HGSOC
MEL
UMAP-1
UMAP-1
B
CD39
C
CD103
D
CD69
E
PD-1
F
2B4
Expression Level
Identity
CD4+ T cells
G
HGSOC
MEL
UMAP-2
UMAP-1
H
CD39
I
CD103
J
CD69
K
PD-1
L
2B4
HGSOC
MEL

Figure S6: Clusters revealed through high dimensional CyTOF comparison of HGSOC and MEL patient CD8+ and CD4+ TILs. Enzymatically digested viably frozen HGSOC (n = 13) and MEL (n = 13) and tumor samples were stained with metal-conjugated antibodies for mass cytometry to characterize the infiltrating CD8+ T cell population. Samples were acquired in batches on a Helios mass cytometer. Panel A shows UMAP of CD8+ TILs for HGSOC and CD8+ TILs for MEL, coloured by the identified 7 clusters. Panel G shows UMAP of CD4+ TILs for HGSOC and CD4+ TILs for MEL, coloured by the identified 12 clusters. Each dot represents 1 cell. Cells are coloured by 7 major clusters. Panels B-F and H-L depict violin plots split per cluster showing the expression level of selected markers (from left to right) CD39 (B and H), CD103 (C and I), CD69 (D and J), PD-1 (E and K) and 2B4 (F and L) identified in CD8+ T cells per tumor type. Cells from HGSOC samples are coloured in red and cells from MEL samples – in green.
