# Supplementary material for: Comparative Analysis of Ovarian Cancer Tumor-Infiltrating T Cells with Those in Melanoma and before and after PD-1 Blockade
Source: Cancer Res Commun. 2026 Nov 12;6(11):2655–68. doi: 10.1158/2767-9764.CRC-26-0011 (PMC13757280; doi:10.1158/2767-9764.CRC-26-0011)
Supplement: Supplementary file 3 [file crc-26-0011_supplementary_table_s1_suppst1.docx]

**Supplementary Table S1: Antibody information for the samples profiled by IHC**.

| **Epitope** | **Fluorochrome** | **Company** | **Cat. No.** | **Clone** |
| --- | --- | --- | --- | --- |
| PanCK | Agilent DAKO | M351501-2 | AE1/AE3 | PanCK |
| MEL Cocktail | Abcam | ab733 | NA | MEL Cocktail |
| CD3 | Roche Diagnostics | 5278422001 | 2GV6 | CD3 |
| CD8 | Roche Diagnostics | 5937248001 | SP57 | CD8 |
| CD137 (4-1BB) | Thermo Fisher Scientific | MA5-13739 | BBK-2 | CD137 (4-1BB) |
| PD-1 | Abcam | ab137132 | EPR4877(2) | PD-1 |
| CD103 | Abcam | ab129202 | EPR4166(2) | CD103 |
| CD163 | Biocare Medical/ Inter Medico (1000096) | BC-CM353AK | 10D6 | CD163 |
| PD-L1 | Abcam | ab228462 | SP142 | PD-L1 |
| CD68 | Agilent | M0814 | KP1 | CD68 |
