# Supplementary material for: Comparative Analysis of Ovarian Cancer Tumor-Infiltrating T Cells with Those in Melanoma and before and after PD-1 Blockade
Source: Cancer Res Commun. 2026 Nov 12;6(11):2655–68. doi: 10.1158/2767-9764.CRC-26-0011 (PMC13757280; doi:10.1158/2767-9764.CRC-26-0011)
Supplement: Supplementary file 4 [file crc-26-0011_supplementary_table_s2_suppst2.docx]

**Supplementary Table S2: Antibody/reagent information for the samples profiled by CyTOF**.

| **Target** | **Tag** | **Company** | **Cat. No.** | **Clone** | **Dilution** |
| --- | --- | --- | --- | --- | --- |
| CD45 | 89Y | BioLegend | 304045 | HI130 | 1:50 |
| CD3 | 115In | BioLegend | 300443 | UCHT1 | 1:25 |
| CD95 | 141Pr | BioLegend | 305631 | DX2 | 1:25 |
| CD45RA | 142Nd | BioLegend | 304143 | HI100 | 1:200 |
| CCR1 | 143Nd | BioLegend | 362902 | 5F10B29 | 1:25 |
| CD38 | 144Nd | BioLegend | 303535 | HIT2 | 1:200 |
| TCRγδ | 145Nd | BioLegend | 331202 | B1 | 1:100 |
| CD14 | 146Nd | BioLegend | 301843 | M5E2 | 1:100 |
| EOMES | 147Sm | eBioscience | 14-4877-82 | WD1928 | 1:50 |
| NKG2D | 148Nd | BioLegend | 320802 | 1D11 | 1:25 |
| FOXP3 | 149Sm | eBioscience | 14-4777-82 | 236A/E7 | 1:200 |
| T-bet | 150Nd | eBioscience | 644825 | B269710 | 1:25 |
| CD8α | 151Eu | BioLegend | 301053 | RPA-T8 | 1:2000 |
| CXCR4 | 152Sm | BioLegend | 306523 | 12G5 | 1:100 |
| CD39 | 153Eu | eBioscience | 14-0399-82 | eBioA1 | 1:200 |
| PD-L1 | 154Sm | eBioscience | 14-5983-82 | MIH1 | 1:25 |
| CD71 | 155Gd | BioLegend | 334102 | CY1G4 | 1:800 |
| CCR5 | 156Gd | BioLegend | 359102 | 2D7 | 1:800 |
| DNAM-1 | 158Gd | BioLegend | 338302 | 11A8 | 1:50 |
| TIM-3 | 159Tb | R&D | MAB2365 | 344823 | 1:200 |
| CD28 | 160Gd | BioLegend | 302902 | CD28.2 | 1:25 |
| HLA-DR | 161Dy | BioLegend | 307651 | L243 | 1:200 |
| CD4 | 162Dy | BioLegend | 300541 | RPA-T4 | 1:2000 |
| CD103 | 163Dy | BioLegend | 350202 | Ber-ACT8 | 1:200 |
| ICOS | 164Dy | BioLegend | 313512 | C398.4A | 1:400 |
| PD-1 | 165Ho | eBioscience | 14-2799-80 | eBioJ105 | 1:50 |
| CD69 | 166Er | BioLegend | 310939 | FN50 | 1:800 |
| CD45RO | 167Er | BioLegend | 304239 | UCHL1 | 1:400 |
| Ki67 | 168Er | BioLegend | 350502 | Ki-67 | 1:2000 |
| 4-1BB | 169Tm | BioLegend | 309802 | 4B4-1 | 1:200 |
| 2B4 | 170Er | BioLegend | 329502 | C1.7 | 1:200 |
| TIGIT | 171Yb | eBioscience | 16-9500-82 | MBSA43 | 1:200 |
| GZMB | 172Yb | eBioscience | MA1-10338 | CLB-GB11 | 1:800 |
| CD8β | 173Yb | eBioscience | 14-5273-82 | SIDI8BEE | 1:1000 |
| CTLA4 | 174Yb | eBioscience | 14-1529-82 | 14D3 | 1:200 |
| GITR | 175Lu | eBioscience | 14-5875-80 | eBioAITR | 1:25 |
| CD31 | 176Yb | BioLegend | 303102 | WM59 | 1:200 |
| DNA1 | 191 Ir | Standard BioTools | 201192A | N/A | 1 nM |
| DNA2 | 193 Ir | Standard BioTools | 201192A | N/A | 1 nM |
| Cisplatin | 195 Pt | Standard BioTools | 201064 | N/A | 1 μM |
| CD25 | 209Bi | BioLegend | 356102 | M-A251 | 1:50 |
