# Supplementary material for: Comparative Analysis of Ovarian Cancer Tumor-Infiltrating T Cells with Those in Melanoma and before and after PD-1 Blockade
Source: Cancer Res Commun. 2026 Nov 12;6(11):2655–68. doi: 10.1158/2767-9764.CRC-26-0011 (PMC13757280; doi:10.1158/2767-9764.CRC-26-0011)
Supplement: Supplementary file 5 [file crc-26-0011_supplementary_table_s3_suppst3.docx]

**Supplementary** **Table S3: Antibody information for the samples profiled by multicolour flow cytometry**.

| **Epitope** | **Fluorochrome** | **Company** | **Cat. No.** | **Clone** | **Dilution** |
| --- | --- | --- | --- | --- | --- |
| Fc Block | N/A | BD Biosciences | 564220 | Fc1 | 1:200 |
| TCRγδ | FITC | eBioscience | 11-9959-42 | B1.1 | 1:50 |
| CD39 | PerCP-eF710 | eBioscience | 46-0399-42 | A1 | 1:100 |
| CD4 | Alexa700 | eBio | 56-0049-42 | RPA-T4 | 1:100 |
| CD3 | BUV395 | BD Biosciences | 563546 | UCHT1 | 1:25 |
| CD19 | BUV737 | BD Biosciences | 741829 | HIB19 | 1:200 |
| fixable viability | eF506 | eBioscience | 65-0866-18 | N/A | 1:800 |
| PD-1 | BV605 | BioLegend | 329924 | EH12.2H7 | 1:100 |
| CD45 | BV650 | BioLegend | 304044 | HI30 | 1:100 |
| CD69 | BV711 | BioLegend | 310944 | FN50 | 1:50 |
| CD8 | SB780 | eBioscience | 78-0088-42 | RPA-T8 | 1:50 |
| CD103 | PE | eBioscience | 12-1038-42 | B-Ly7 | 1:100 |
| 2B4 | PE-Dazzle | BioLegend | 329522 | C1.7 | 1:200 |
