# Supplementary material for: Comparative Analysis of Ovarian Cancer Tumor-Infiltrating T Cells with Those in Melanoma and before and after PD-1 Blockade
Source: Cancer Res Commun. 2026 Nov 12;6(11):2655–68. doi: 10.1158/2767-9764.CRC-26-0011 (PMC13757280; doi:10.1158/2767-9764.CRC-26-0011)
Supplement: Supplementary file 6 [file crc-26-0011_supplementary_table_s4_suppst4.docx]

**Supplementary Table S4: Clinical characteristics of melanoma patients**

| **Sample ID** | **Tumor type** | **Cohort** | **Histologic type** | **Sex** | **Age at surgery** | **Stage** | **Prior**  **treatment** | **Mutations** |
| --- | --- | --- | --- | --- | --- | --- | --- | --- |
| Mel-1 | Melanoma | IHC | Cutaneous (lentigo maligna) | M | 77 | III | No | BRAF, NRAS |
| Mel-2 | Melanoma | IHC, CyTOF | Cutaneous | M | 74 | IV | No | BRAF V600E/K mutation |
| Mel-3 | Melanoma | IHC | Mucosal | M | 55 | III | No | BRAF, cKIT exon 17 mutation |
| Mel-4 | Melanoma | IHC, CyTOF | Mucosal | M | 69 | III | No | NRAS |
| Mel-5 | Melanoma | IHC, CyTOF | Cutaneous | M | 59 | III | No | BRAF V600E/K |
| Mel-6 | Melanoma | IHC, CyTOF | Cutaneous | M | 57 | III | No | BRAF V600E |
| Mel-7 | Melanoma | IHC | Cutaneous | M | 59 | IV | No | BRAF, NRAS, |
| Mel-8 | Melanoma | IHC | Cutaneous | F | 54 | III | No | none |
| Mel-9 | Melanoma | IHC | Cutaneous (lentigo maligna) | M | 80 | III | No | 2 atyp BRAF |
| Mel-10 | Melanoma | IHC | Acral | M | 60 | IV | No | cKIT exon 11 |
| Mel-11 | Melanoma | IHC | Unkown primary | F | 63 | III | No | BRAF, NRAS, |
| Mel-12 | Melanoma | CyTOF | Metastatic | M | 53 | III | No | NRAS |
| Mel-13 | Melanoma | CyTOF | Invasive Spindle Cell, Amelanotic | M | 84 | UKN | No | UKN |
| Mel-14 | Melanoma | CyTOF | Malignant, nodular | M | 72 | UKN | No | None |
| Mel-15 | Melanoma | Flow | Cutaneous | M | 42 | IVB | No | NRAS |
| Mel-16 | Melanoma | Flow | Unknown primary | M | 40 | IVC | Ipilimumab, Pembrolizumab | None |
| Mel-17 | Melanoma | Flow | Cutaneous | F | 46 | IVB | Interferon, Ipilimumab, Vemrafemib/Trametib, Pembrolizumab | BRAF |
| Mel-18 | Melanoma | Flow | Cutaneous | F | 61 | IVC | Nivolumab, DAB/TRAM | BRAF |
| Mel-19 | Melanoma | Flow | Cutaneous | M | 61 | IVC | IPI/NIVOx4, Nivolumab, Cobimetinib | BRAF |
| Mel-20 | Melanoma | Flow | Uveal | F | 46 | IVC | Pembrolizumab, Tebentafusp | BAP1 |

M: Male; F: Female; UKN: unknown
